# Potential Toxicity Evaluation of Protopine in *Macleaya cordata* (Willd.) R. Br.—A Bioactivity Guided Approach

**DOI:** 10.3389/fvets.2021.752767

**Published:** 2021-11-25

**Authors:** Wanjun Hu, Fan Yang, Weixue Liu, Liyang Guo, Liwen Ai, Xiaomeng Zhang, Zunlai Sheng, Chunbo Gao

**Affiliations:** ^1^College of Veterinary Medicine, Northeast Agricultural University, Harbin, China; ^2^Heilongjiang Key Laboratory for Animal Disease Control and Pharmaceutical Development, Northeast Agricultural University, Harbin, China; ^3^English Department, Heilongjiang College of Foreign Languages, Harbin, China

**Keywords:** *Macleaya cordata* (Willd.) R. Br., LD50, protopine, bioactivity-guided fractionation, H&E

## Abstract

*Macleaya cordata* (Willd.) R. Br. (*M. cordata*) is a perennial herb known for its chemotherapeutic properties, strong feeding additive, and potential antidiarrheal drug. Despite its therapeutic potentials, its clinical applications are hindered by an apparent lack of toxicity data. In this study, the toxic ingredients of this plant were investigated using a bioactivity-guided approach. Two compounds, protopine and allocryptopine, were purified and elucidated by LC-MS, ^1^H-NMR, and ^13^C-NMR. Protopine, a primary component in *M. cordata*, had an LD_50_ of 313.10 mg/kg i.e., which was considered toxic. An autopsy was performed on protopine-administered mice, and the histopathology of the kidney, liver, brain, heart, lung, and spleen was determined. Autopsy findings included hemorrhage in the respiratory system, lung congestion, and hemorrhage and edema in the parenchymatous organs (heart, liver, kidney, and brain). Histopathology confirmed the pathological changes in the brain, liver, and kidney. Protopine is one of the principal bioactive constituents of many phytopreparations used in veterinary and human medicine, such as Sangrovit and Iberogast. Our findings indicated that phytopreparations containing protopine might pose a serious health threat to humans and animals.

## Introduction

The abuse of veterinary antibiotics has long attracted worldwide attention (1). Misapplication or illegal use of veterinary medicine and contraband medicines may result in harmful residues to the health of animals and humans (2). China has a vast population and a large agricultural sector; thus, it is the world's largest producer and consumer of antibiotics (3). One of the major problems faced by humans is the increase in antibiotic resistance. Therefore, the European Union banned the use of antibiotics in animal feeds in 2006 (4), and China prohibited the use of growth-promoting antibiotics (except traditional Chinese medicine products) in feeds in 2020 (5). Therefore, the use of safe and efficient natural medicine in feed additives has attracted attention worldwide.

Traditional Chinese medicine (TCM) has been used in healthcare to treat various diseases for thousands of years in China and other eastern Asian countries (6). At present, TCM remains a popular choice for consumers due to its unique efficacy and mild side effects (7). Unlike traditional medicine, chemical medicines have adverse effects and clinical problems (8). However, incident reports of TCM toxicity have been occasionally reported in recent years, leading to TCM facing significant challenges (9). The concept of toxicity in ancient China differs from modern medicine, as modern medicine includes both pharmacological and toxicological considerations (6). The first monograph of TCM was Shennong Bencaojing, which was divided into three categories based on the “toxic” grade: top grade, average grade, and lowest grade (10). According to ancient healers, all drugs contain toxicity, while all toxins can be medicines. Although certain TCM herbal products are toxic, TCM is still widely prescribed in clinics. Toxic TCM can have a prominent effect when used rationally, but excessive use generally leads to severe organ damage or death.

*Macleaya cordata* (Willd.) R. Br. (*M. cordata*) is a traditional Chinese herb that belongs to the genus *Macleaya* and the family *Papaveraceae* (11). It is used to treat inflamed wounds, cervical cancer, thyroid cancer, relieve muscle pain, and bee stings (12–14). Some of the *M. cordata* extract products such as Sangrovit® and Sensopower® have been developed and used in veterinary medicine and agriculture due to its wide range of pharmacological activities (15). In addition, it may be used as a plant insecticide (11). The *M. cordata* extract is an antibiotic alternative that does not promote the spread of antibiotic resistance genes (5). *M. cordata* is a highly toxic TCM first recorded in Ben Cao Gang Mu Shi Yi. It is mainly used to treat various skin diseases and is not administered orally. Animal poisoning and even death after oral or intramuscular injection of *M. cordata* extract products have been reported in China. However, there are inadequate toxicology studies on *M. cordata* extracts and products. The *M. cordata* is China's first class-II veterinary medicine. Therefore, it is critical to identifying the primary toxic component in *M. cordata* extracts.

This study aimed to isolate and identify the toxic compounds in *M. cordata* extracts through bioactivity-guided fractionation. This study found that protopine was the primary toxic component in *M. cordata* extracts, and the LD_50_ and acute toxicity were evaluated in an experimental ICR mouse model. Interestingly, protopine is one of the main active alkaloids in *M. cordata* and is one of the main active ingredients in other medicinal plants. This is the first study on the toxicity of protopine. These findings offer a useful reference for assessing and evaluating the toxic properties of *M. cordata*.

## Materials and Methods

### Plant Material and Chemicals

The root of *M. cordata* (Willd.) R. Br. was collected from Sanmenxia City, Henan Province, China (N34°31′24″, E111°21′42″), in September 2020 and was authenticated by Dr. Junkai Wu (Heilongjiang University of Traditional Chinese Medicine). A voucher specimen (No. 20150601) was deposited in the Herbarium of Northeast Agricultural University.

Silica gel GF254 plates and silica gels (200–300 mesh) were obtained from Qingdao Marine Chemical, Inc., Qingdao, China. Methanol and formic acid were of HPLC grade (Hangzhou Reagent Company). The other analytical grade reagents were purchased from Komil Chemical Reagents Co., Ltd. (Tianjin, China).

### Extraction and Toxicity-Guided Fractionation of *M. cordata*

*M. cordata* (5.0 kg) was extracted with 95% ethanol at 80°C for 2 h. After filtration, the filtrate was evaporated under reduced pressure to yield a dry extract (EtOH extract, yield 11%). The dried ethanol extract (500 g) was suspended in H_2_O, then partitioned with dichloromethane (DCM), ethyl acetate (EA), and n-butyl alcohol (n-BuOH) successively. Each fraction was evaluated to screen for the toxic fraction.

The n-BuOH fraction (192.5 g) was separated on a silica gel column (100–200 mesh) and eluted with DCM, as well as mixtures of DCM-MeOH (from 95:5 to 1:1) to obtain five subfractions (Fr. A-E). The Fr. D (85 g) was further divided into three subfractions (D1–D3) using a step gradient of the PE–EtOAc–triethylamine solvent system (3:1:0.05 to 0:1:0.05). Compound 1 (8.4 g) and compound 2 (4.2 g) were separated from subfraction D2. Each fraction was evaluated to screen for the toxic fraction.

**Figure 1:**
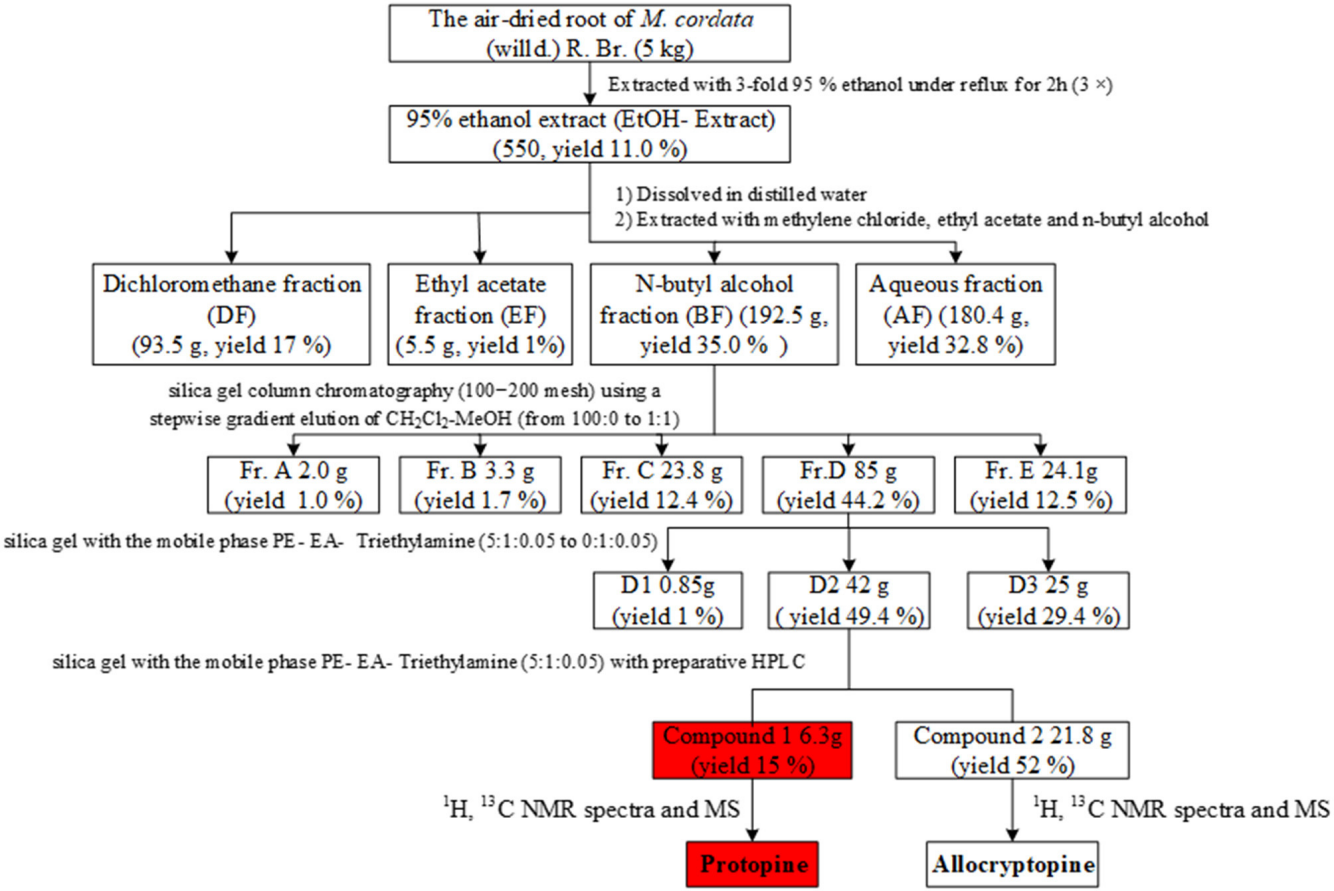
Extraction and fractionation of the root of *M. cordata* (Willd.) R. Br.

The extract and fractionation process is shown in Figure 1. Subfraction D2 was the most poisonous substance after toxicity tests on subfractions, and its major components were identified using LC-MS, ^1^H-NMR, and ^13^C-NMR.

### Toxicity Assay of *M. cordata* Extracts

#### Animals

Seven-week-old male and female ICR mice (20.0 ± 2.0 g) were purchased from the Experimental Animal Center of Harbin Medical University (Harbin, China). The feeding and environmental conditions were provided according to the regulations and ethics of the Northeast Agricultural University Animal Ethics Committee (approved protocol number: SRM-06).

### Oral Toxicity Tests in Mice

#### Oral Toxicity Studies of *M. cordata* Ethanol Extracts

Male ICR mice were acclimatized for 1 week in a controlled environment at 25 ± 2°C, 50–60% humidity, and a 12:12-h light/dark cycle with free access to food and water. A total of 42 male mice were divided into seven groups (six mice per group). The mice were weighed, and the doses were calculated based on their body weight. The ethanol extract (EE) was suspended in water. Each group received administration of either normal saline or protopine (5, 4, 2, 1, 0.5, and 0.25 g/kg i.g) at 10 ml/kg. The clinical effects and mortality of mice were continuously observed during the first 2 h after administration of EE. Following that, the mice were observed every 8 h for 24 h, then daily for 14 days. Autopsies were performed to determine the exact cause of the dead mice. After 14 days, surviving mice were killed by cervical dislocation under ether anesthesia for macroscopic observation.

#### Oral Toxicity Studies of All Isolates

Sixty-six 7-week-old male ICR mice were acclimatized for 1 week and fasted overnight before the experiment. The acute oral toxicity of all isolates was evaluated in ICR mice. The general behavioral changes of the mice were systematically recorded, with individual records maintained for each animal. Autopsies were performed to determine the exact cause of the dead mice. The brain, livers, spleen, kidneys, hearts, and lungs of the mice were weighed.

### Toxicity Assay of Protopine

#### LD_50_ of Protopine by Oral Gavage in Mice

Male and female ICR mice (n = 30, n = 30) were randomly divided into six experimental groups of 10 animals each, with each receiving administration of either normal saline or protopine (833, 500, 300, 180, and 108 mg/kg i.g) in a volume of 10 ml/kg. Protopine was dissolved in glacial acetic acid (0.5%). The mice were monitored for signs of toxicity or mortality for an additional 14 days. Autopsies were performed to determine the exact cause of death in the poisoned mice. On day 15, the surviving mice were killed by cervical dislocation under anesthesia for macroscopic observation. The brain, livers, spleen, kidneys, hearts, and lungs of the mice were weighed during autopsy. Relative organ weights were based on the organ-to-body-weight ratio. A small part of organ tissues was preserved in 10% neutral-buffered formalin to identify any histopathological changes. The fixed organs were embedded in paraffin, sectioned, and stained with hematoxylin and eosin (H&E). LD_50_ was determined using the method described by Miller and Tainter (16).

### Statistical Analysis

All data were expressed as mean ± SD. Statistical comparisons were analyzed by two-way ANOVA of multiple comparisons using GraphPad Prism 7. In all the tests, *p* < 0.05 was considered to be statistically significant.

## Results

### Screening of Toxic Components

#### Toxicity Effects of the Ethanol Extracts of *M. cordata*

Table 1 displays the mortality and time of death due to the administration of EE in the experimental groups. The lethal dose for mice was discovered to be 5 g; there were no deaths at 0.25 g.

**Table 1:** Acute toxicity effect of ethanol extract of *M. cordata* (EE) in mice.

**Groups**	**Does (g/kg)**	**Number of death**	**Death of time**			
			**0–10 min**	**10–30 min**	**30–0 min**	**1–2 h**
1	0.25	0/6				
2	0.5	1/6	1			
3	1	2/6	1			1
4	2	3/6	1	2		
5	4	5/6	4	1		
6	5	6/6	6			

The obvious toxic symptoms were observed in mice treated with EE at 0.5, 1, 2, 4, and 5 g/kg. Clinical signs of poisoning in mice administered i.g. with a lethal dose of EE was as follows: within seconds of oral EE administration at 4–5 g/kg, mice exhibited signs of muscular weakness and severe ataxia. Within 1 min, poisoned mice developed muscle tremors and spastic involuntary muscular contractions, as evidenced by a marked extension of the head, neck, and tail flicking and signaling. These involuntary tremors became more severe until the mice were fatigued and unable to move voluntarily. Lethally poisoned mice struggled for breath, resulting in spasmodic jumps and shaking. These animals quickly became cyanotic and died. Lethally poisoned animals typically died within 10 min of treatment. Within seconds of oral EE administration at 0.5–2 g/kg, the mice were excited and restless, jumping and running in circles, scratching their faces, and erecting their tails. Following that, the mice showed mental depression, slow breathing, closed eyes, coma, muscle tremors, and spastic involuntary muscular contractions within 5 min. These animals eventually became cyanotic and died. The surviving animals recovered within 24 h. After administration of EE for 14 days, there were no behavioral changes or mortality in the treated mouse groups.

An autopsy revealed that mice receiving lethal doses of EE had congested vital organs (heart, brain, liver, kidney, lung) without any signs of damage or necrosis. Administration of EE for 14 days resulted in flatulence and pathological changes in the fatty liver of the high-dose-treated mouse groups. The vital organs in the control mouse group were normal.

#### Toxicity Effects of All Isolates of the EE

The first four fractions were obtained using bioactivity-guided fractionation: dichloromethane fraction (DF), ethyl acetate fraction (EF), n-butyl alcohol fraction (BF), and aqueous fraction (AF). The acute toxicity results of the four fractions, including the mortality and time of death of mice in the DF, EF, BF, and AF groups, are shown in Table 2. The lethal dose for BF was 1 g. In the BF group, the mice displayed muscular weakness, severe ataxia, tremors, convulsions, lethargy, sleep, coma, and mortality. Lethally poisoned animals typically died within 10 min of treatment with BF. However, there were no deaths in the DF, EF, and AF groups.

**Table 2:** Acute toxicity effect of different polarity fractions of EE in mice.

**Group**	**Does (g/kg)**	**Number of death**	**Deathy of time (min)**
MF	1	0/6	
EF	1	0/6	
BF	1	6/6	0 - 10
AF	1	0/6	

The autopsy on BF group mice showed congestion in the vital organs: heart, brain, liver, kidney, and lung. However, these vital organs were normal in the DF, EF, and AF groups.

**Figure 2:**
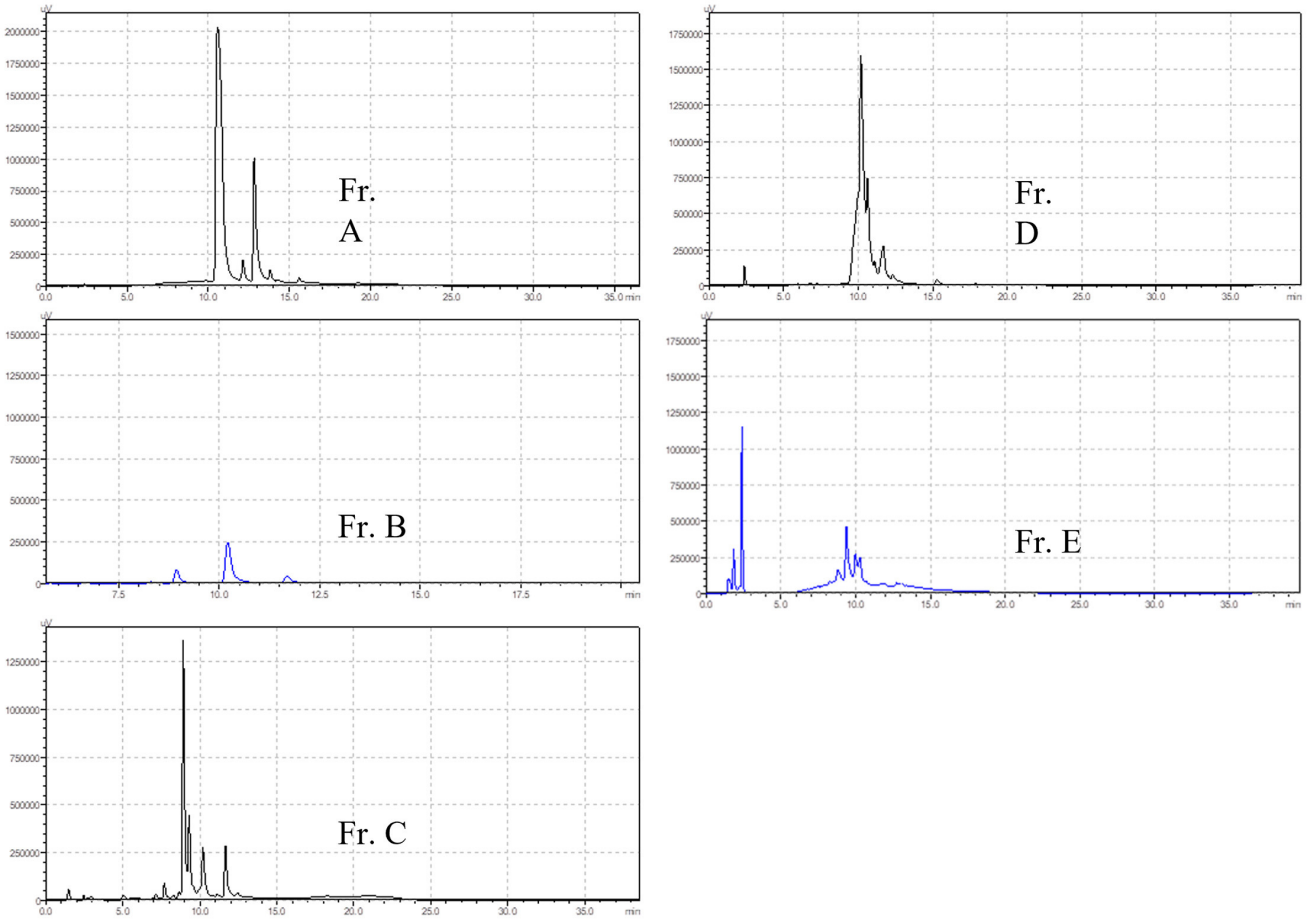
HPLC chromatograms of different polarity fractions of *M. cordata* (EE) ethanol extracts.

BF was then separated by silica gel column chromatography, yielding five fractions (Fr. A–E). The chromatograms of the five fractions were analyzed by HPLC, as shown in Figure 2. Acute toxicity tests of the five subcomponents were carried out in mice, and the results are displayed in Table 3. The mortality and time of death of mice due to the administration of Fr. A, B, C, D, and E are reported. The results revealed that one-half of all deaths in mice treated with Fr. D at 0.5 g/kg occurred within 30 min. One mouse died after being treated with Fr. C at 0.5 g/kg for 8 h. There were no deaths when mice were administered with Fr. A, B, and E.

**Table 3:** Acute toxicity effect of different subfractions of BF in mice.

**Group**	**Does (g/kg)**	**Number of death**	**Deathy of time**		
			**0–10 min**	**10–30 min**	**1–8 h**
Fr. A	0.5	0/6			
Fr. B	0.5	0/6			
Fr. C	0.5	1/6			1
Fr. D	0.5	3/6	2	1	
Fr. E	0.5	0/6			

The clinical signs of poisoning in mice treated with Fr. C and D were similar to mice treated with BF. There were no significant differences based on autopsies of the dead mice. Autopsies revealed that the tissue, heart, brain, liver, kidney, and lung were congested, and there was hepatic edema in dead mice that were administered with Fr. C and D. All mice that were administered Fr. A, B, and E survived without obvious poisoning symptoms.

The Fr. D was subsequently separated by silica gel column chromatography, yielding three fractions (D1–3). Acute toxicity tests for the three Fr. D subcomponents were carried out in mice, and the results are displayed in Table 4. The mortality and time of death of mice due to the administration of D1, D2, and D3 are reported. The lethal dose for D2 was discovered to be 0.5 g. One mouse died in each of the groups administered with D1 and D3, respectively. Mice that were administered with DR displayed obvious clinical signs of poisoning.

**Table 4:** Acute toxicity effect of different subfractions of Fr. D in mice.

**Group**	**Does (g/kg)**	**Number of death**	**Deathy of time**			
			**0–10 min**	**10–30 min**	**1–8 h**	**8–24h**
D.1	0.5	1/6			1	
D.2	0.5	6/6	4	1	1	
D.3	0.5	1/6				1

**Figure 3:**
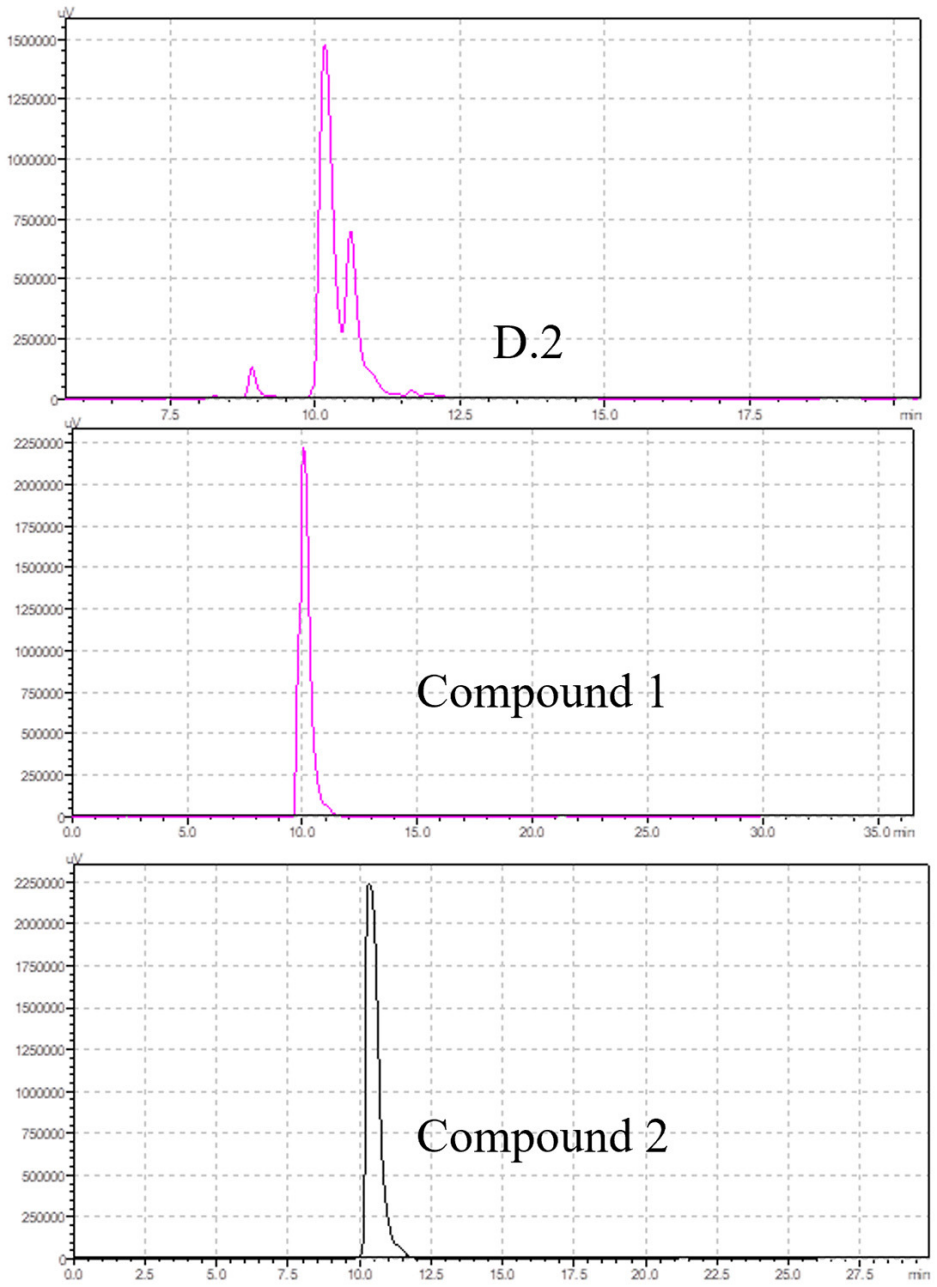
Chromatograms of D2, compound 1, and compound 2 by HPLC.

The study found that the D2 fraction was the main toxic substance base of *M. cordata*. Compounds 1 and 2 were isolated and purified by silica gel column chromatography from D2. The chromatograms of compounds 1 and 2 and D2 were analyzed by HPLC, as shown in Figure 3. The retention times for compounds 1 and 2 were around 10.045 and 10.328, respectively. Acute toxicity tests of compounds 1 and 2 were carried out in mice, and the results are displayed in Table 5. The mortality and time of death due to compounds 1 and 2 are as follows: five mice died within 60 min of being treated with compound 1 at 0.5 g/kg. One mouse died after 60 min of compound 2 treatment at 0.5 g/kg. The results demonstrated that compound 1 was the primary toxic component in *M. cordata* extracts, while compound 2 had mild toxicity. Compound 1 induced less mortality in mice than a mixture of D2 at the same dose. The results suggest that compounds 1 and 2 could cause combined intoxication.

**Table 5:** Acute toxicity effect of different subfractions of D.2 in mice.

**Group**	**Does (g/kg)**	**Number of death**	**Deathy of time**	
			**0–10 min**	**30–60 min**
Compound 1	0.5	5/6	4	1
Compiund 2	0.5	1/6		1

### Identification of Compounds 1 and 2

**Figure 4:**
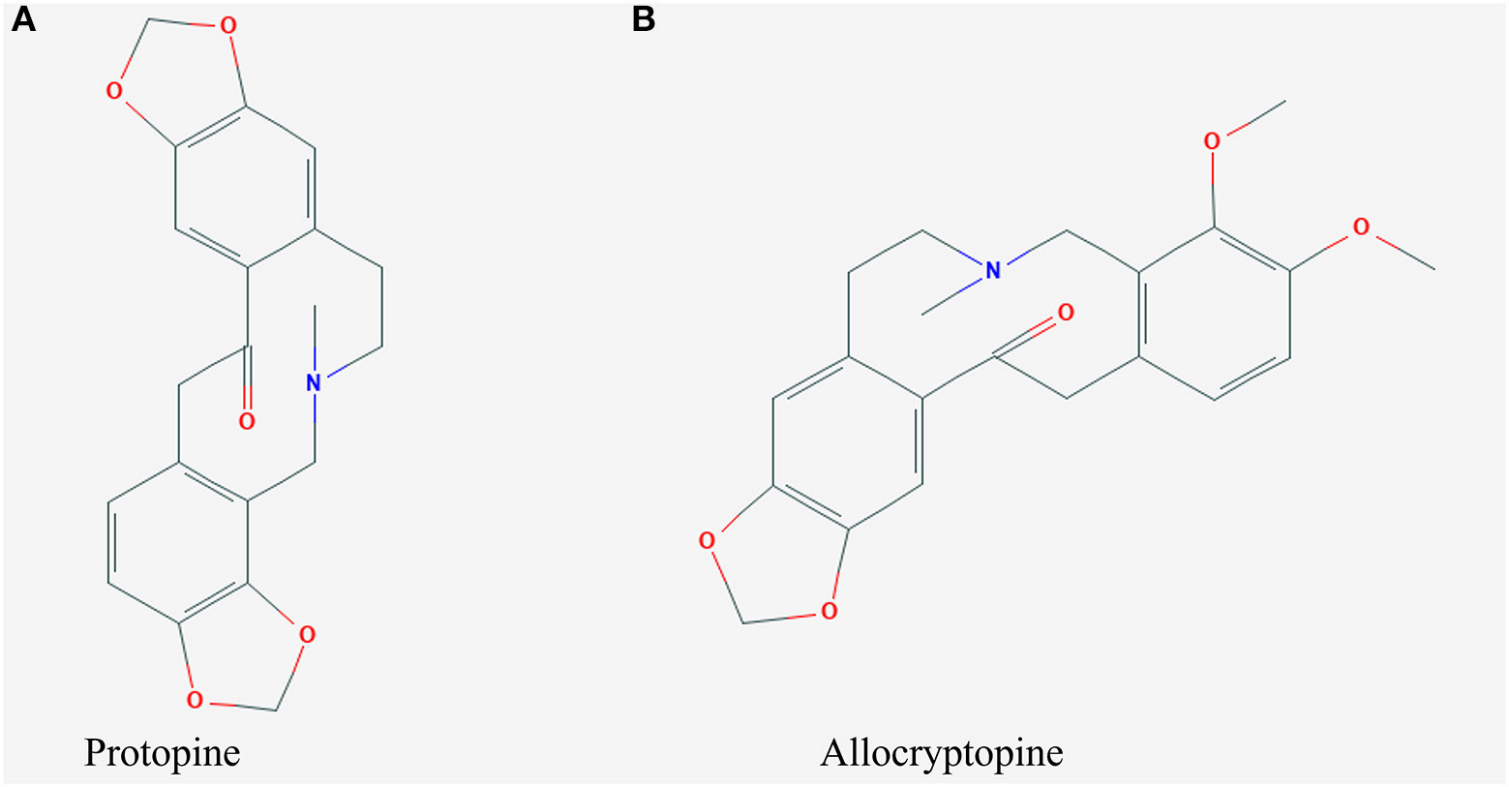
Chemical structures of **(A)** protopine and **(B)** allocryptopine.

Compound 1 was obtained in the form of a white powder. Its molecular formula was determined to be C_20_H_19_NO_5_ based on the HRESIMS ([M+H]^+^ m/z 354.1316, calculated C_21_H_20_O_11_Na, 354.3832). ^1^H-NMR (CDC_l3_, 600 MHz) δ: 6.925 (1H, s, H-1), 6.709 (1H, d, J = 7.8 Hz, H-12), 6.681 (1H, d, J = 7.8 Hz, H-11), 6.662 (1H, s, H-4), 5.968 (2H, s, -OCH_2_O-), 5.943 (2H, s, -OCH_2_O-), 3.630 (6H, br s), 2.660 (4H, br s), 1.972 (3H, s, N-CH_3_). ^13^C-NMR (150 MHz, DMSO-d_6_) δ: 194.2 (C-14), 148.0 (C-3), 146.3 (C-2), 146.0 (C-9), 145.9 (C-10), 135.9 (C-4a), 132.5 (C-14a), 128.8 (C-12a), 125.0 (C-12), 117.7 (C-8a), 110.4 (C-4), 108.1 (C-1), 106.8 (C-11), 101.2 (2, 3-OCH_2_O-), 100.9 (9, 10-OCH_2_O-), 57.7 (C-6), 51.0 (C-8), 46.2 (C-13), 41.5 (N-CH_3_), 31.6 (C-5). The chemical structure of protopine is shown in Figure 4A.

Compound 2 was also obtained as a white powder. Its molecular formula was determined to be C_21_H_23_NO_5_ based on the HRESIMS ([M+H]^+^ m/z 370.1605, calculated C_21_H_24_ NO_5_, 370.4262). ^1^H-NMR (DMSO-d6, 600 MHz) δ:6.956 (1H, s, H-1), 6.930 (1H, d, J = 8.0 Hz, H-11), 6.874 (1H,d, J = 8.0 Hz, H-12), 6.796 (1H, s, H-4), 5.997 (2H, s,-OCH2O-), 3.776 (3H, s, -OCH3), 3.655 (3H, s, -OCH3), -CH2- [δH2.3614~δ3.2367 (4H, brs), δ3.2367~δ3.3487 (2H, brs), δ3.7266 (2H, s)],1.859 (3H, s, N-CH_3_). ^13^C-NMR (150 δMHz, DMSO-d_6_) δ: 110.612 (C-1), 135.584 (C-2), 30.646 (C-3), 57.243 (C-4), 50.973 (C-5), 128.823 (C-6), 151.361 (C-7), 146.864 (C-8), 108.311 (C-9), 128.026 (C-10), 129.611 (C-11), 45.563 (C-12), 172.615 (C-13), 132.593 (C-14), 111.188 (C-15), 145.947 (C-16), 101.537 (C-17), 147.730 (C-18), 41.392 (C-19), 60.641 (C-20), 55.940 (C-21). The chemical structure of allocryptopine is shown in Figure 4B.

### Determination of LD_50_ for Protopine

The LD_50_ of protopine in mice was determined to be 313.10 mg/kg (245.26–397.17, 95% confidence interval) after oral administration, which is considered toxic. The results of oral protopine administration in mice, including mortality due to protopine administration in the experimental groups, are shown in Table 6. The lethal dose was 833.3 mg, but there were no deaths when the dose was reduced to 108 mg. Mice treated with protopine displayed very similar clinical poisoning symptoms to mice treated with a mixture of isolates. The timings of death due to the administration of protopine are reported in Table 7. The lethally poisoned animals typically died within 10 to 30 min of treatment. After 2 days of oral administration, no mice died. In addition, the mice showed no abnormalities in movement, feeding, and drinking after 14 days of oral protopine administration.

**Table 6:** Results of the lethal doses of protopine for the determination of the LD_50_ after oral administration in mice (*n* = 10).

**Group No**.	**Does (mg/kg)**	**Log dose**	**Deaths number**	**Deaths (%)**	**95% confidence interval**
0	0		0/10	0	245.26–397.17
1	108	2.03	0/10	0	
2	180	2.26	1/10	0.1	
3	300	2.48	4/10	0.4	
4	500	2.70	9/10	0.9	
5	833.3	2.92	10/10	1	

**Table 7:** The death time of protopine—induced in mice.

**Group no**.	**death of time**					
	**0–10 min**	**10–30 min**	**0.5–2 h**	**2–8 h**	**8–24 h**	**24–48 h**
0						
1						
2		1				
3	1	2	1			
4	5	1		1	2	
5	7	1	1	1		

On autopsy and histopathological examination, there were no significant differences between the mice receiving lethal doses of protopine. The changes observed during the autopsy were mainly hemorrhage in the respiratory system (lung), congestion, hemorrhage, and edema in the parenchymatous organs (heart, liver, kidney, and brain).

**Figure 5:**
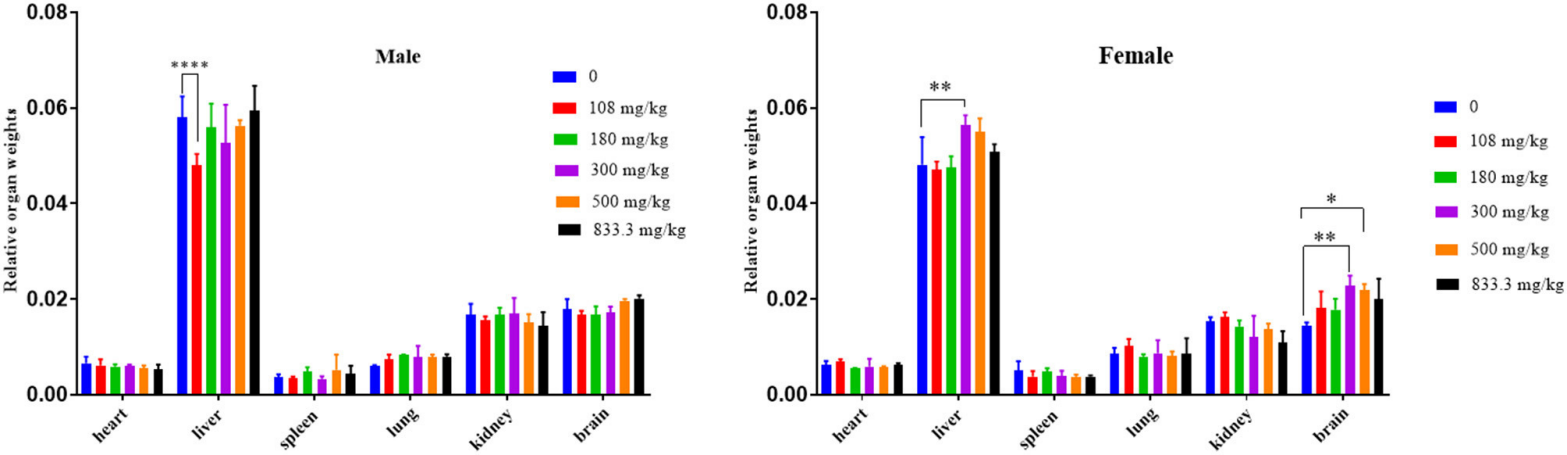
Changes of relative organ weights in male and female mice exposed to different doses of protopine at 0, 108, 180, 300, 500, and 833.3 mg/kg. *p < 0.05, **p < 0.01, ****p < 0.001.

In this study, there was no significant difference in the body weight of the surviving mice. In protopine-treated groups of female mice, a dose-dependent increase in relative organ (liver and brain) weight was observed (Figure 5). The relative brain weights of the 500-mg/kg (*p* < 0.01) and 300-mg/kg groups (*p* < 0.05) were significantly increased compared to the control group (0), indicating that protopine induced brain edema after cerebral hemorrhage in female mice. The relative liver weights of the 300-mg group were significantly increased compared to the control group (0) (*p* < 0.01), indicating that protopine induced liver edema after cerebral hemorrhage in female mice. In male mice, it was notable that the relative liver weights in the 108-mg/kg group were significantly decreased compared to the control group (0).

**Figure 6:**
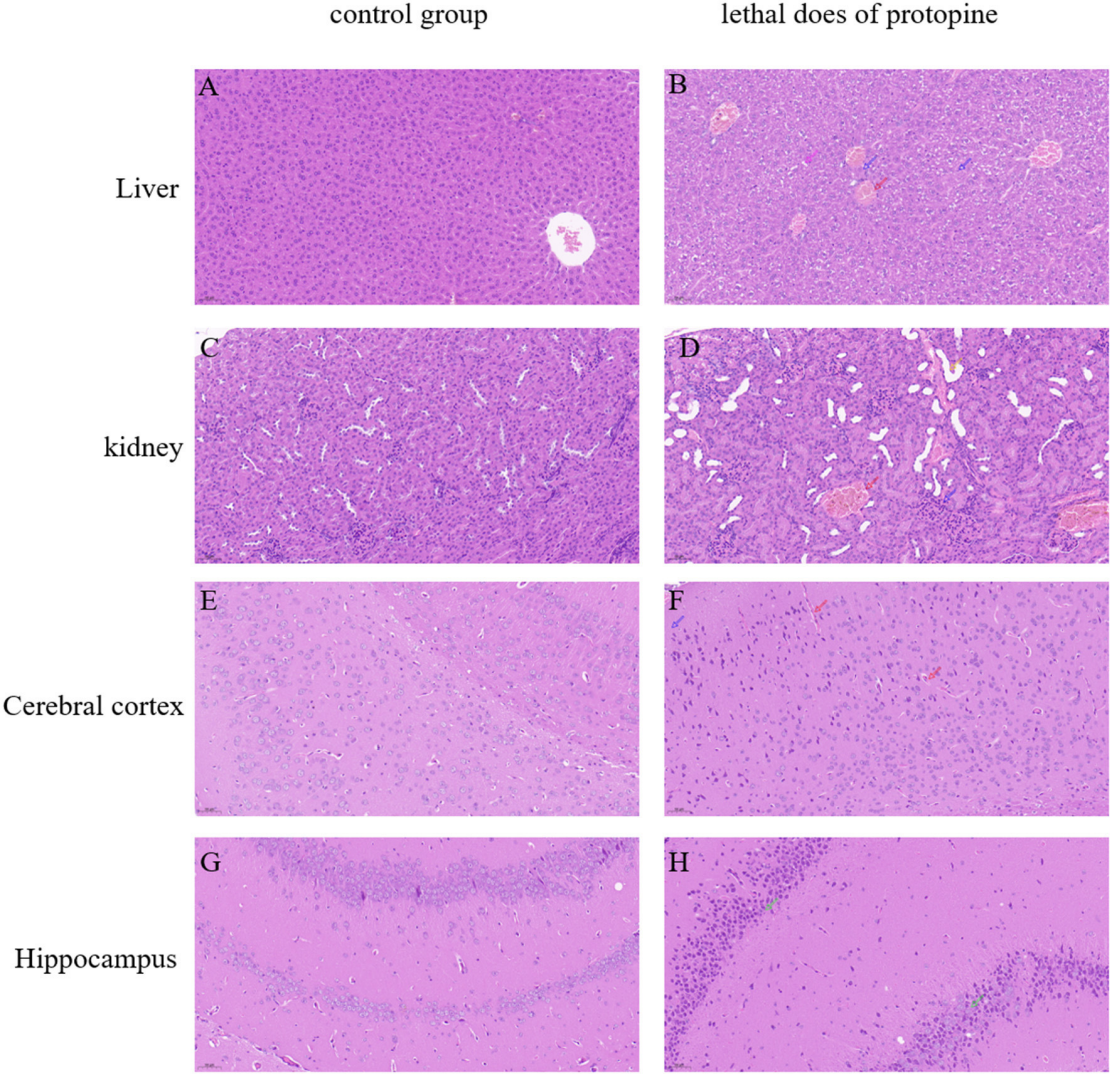
Microscopic analyses of liver, kidney, cerebral cortex, and hippocampal tissue sections obtained from normal **(A,C,E,G)** and protopine-treated mice **(B,D,F,H)**. Tissues were stained with HandE and observed under a microscope at ×200 magnification. The scale bar represents 50 μm. **(A)** Normal liver tissue; **(B)** vacuolar degeneration (pink arrow), nuclear pyknosis (blue arrow), and hyperemia in the central vein (red arrow); **(C)** normal kidney tissue; **(D)** the glomerulus showed increased cellular composition (blue arrow), medium dilatation of the renal tubules, flattened epithelial cells of the renal tubules (yellow arrow), and congested and dilated vasculature (red arrow); **(E)** normal cerebral cortex tissue; **(F)** capillary congestion dilated of meninges (red arrow), edema, and loose structure in the superficial layer of the cerebral cortex, and few nerve cells seen with pyknotic hyperchromatism (blue arrow); (G) normal hippocampus tissue; and **(H)** significant nerve cell necrosis, as well as nucleus pyknosis and fragmentation in the hippocampus (green arrows).

The H&E experiment further validated the pathological changes (Figure 6). Histological examination was performed on the liver, kidney, and brain of the protopine-treated dead and surviving mice. There was no significant difference in tissue sections of the dead mice after protopine treatment. However, histopathological changes were observed in the liver, kidney, and brain (Figure 6). A normal liver's “control” section displayed a typical liver structure, with a single radial arrangement centered on the central vein (Figure 6A). Evident changes, including vacuolar degeneration, nuclear pyknosis, and hyperemia in the central vein, were observed in the liver tissues of mice treated with lethal doses of protopine (Figure 6B). The “control” section of a normal kidney showed that the renal medulla was orderly, and no pathological changes were observed (Figure 6C). The renal tissue of mice treated with lethal doses of protopine displayed increased cellular composition of the glomerulus, medium dilatation of the renal tubules, flattened epithelial cells of the renal tubules, and congested and dilated vasculature (Figure 6D). All cortex and brain tissue cells were arranged regularly in the control group, with the cytoplasm and nuclei stained uniformly (Figure 6E). The cerebral cortex tissue of mice treated with lethal doses of protopine displayed capillary congestion dilated of meninges, edema, and loose structure in the superficial layer of the cerebral cortex, and fewer nerve cells with pyknotic hyperchromatism (Figure 6F). The hippocampal neurons were arranged regularly in the control group (Figure 6G). The hippocampus of mice treated with lethal doses of protopine displayed significant nerve cell necrosis, as well as nucleus pyknosis and fragmentation (Figure 6H).

## Discussion

According to Yang et al. (17), *M. cordata* extracts have been widely used as a feed additive in mammals, poultry, and fish. *M. cordata* is a highly toxic medicine plant that swallowed in large doses by livestock can lead to death according to the Pharmacopeia of the People's Republic of China 2015. However, the toxicology and toxic target organ of *M. cordata* extracts are poorly understood. Therefore, the toxic ingredients of this plant were investigated based on mortality of mice using the bioactivity-guided approach. In medicine plants, the bioassay-guided fractionation technique is widely used to screen and identify bioactive fractions (18–20). In this study, protopine and allocryptopine were identified as the toxic substances in *M. cordata* based on mortality of mice. Besides, protopine was the primary toxic component, while allocryptopine was the low toxic component. In mice, the toxicity effect of combined protopine and allocryptopine administration was stronger than that of protopine alone. The finding demonstrates that there is potential that the toxicosis elicited by one toxin could potentiate the toxicity of another toxin within the same plant (21).

The preliminary screening of primary toxic target organs based on the mouse behavioral changes, organ weight (absolute and relative) changes, autopsy, and H&E experiments. According to Liu et al. (22), protopine and allocryptopine were the main pharmacological components of *M. cordata*. These findings suggest that *M. cordata* may pose a serious threat to human and animal health. The mice treated with the toxic *M. cordata* extracts exhibited signs of muscular weakness. This finding suggests that the administration of *M. cordata* extracts induces neurotoxicity. The results of the H&E experiment further validated pathological damage in the brain of mice induced with the *M. cordata* extracts. Although *M. cordata* extracts have excellent pharmacological activity, they are also toxic (23). Based on the autopsy, flatulence and fatty liver degeneration were observed in the mice induced with the toxic protopine and allocyptopine. This finding suggests that the administration of *M. cordata* extracts induces gastrointestinal diseases and hepatotoxicity in mice. An increasing number of studies have reported that gut microbiota is closely related to human health (24). Gut microbial communities are involved in neurological diseases by modulating the microbiota–gut–brain axis (24). Studies have shown that drug-induced liver injury is linked to gut microbiota dysbiosis (25, 26). These findings led to the hypothesis that exposure to *M. cordata* extracts disrupts the diversity and abundance of the gut microbiome, resulting in metabolic diseases, alimentary canal diseases, and even neurotoxicity. The present study gives a novel insight and method on toxic ingredients and target organ screening for highly toxic medicinal plant.

The first step in assessing and evaluating drug toxicology is the determination of LD_50_ (27). The LD50 of protopine, the primary toxic component in *M. cordata*, was 313.10 mg/kg i.g., which was considered toxic, using the Miller and Tainter method. The necropsy results showed that protopine induced toxicological effects on the different main organs (heart, liver, kidney, and lung) of mice, as well as their nerves. Protopine not only has been shown to effectively cross the blood–brain barrier but also is rapidly and widely distributed in various tissues, with the highest protopine levels detected in the small intestine, followed by the stomach and kidney, lasting 8 h following administration in mice (28). This may be a reasonable explanation for how protopine exposure causes neurotoxicity, gastrointestinal diseases, and toxic effects on various main organs. Under the same conditions, the acute toxicity of protopine showed that the mortality of female mice was higher than that of males. These results suggest that protopine has sex-dependent differences in toxicity. Previously, it has been demonstrated that protopine may be metabolized in the intestinal tract and undergo enterohepatic circulation. Besides, the biotransformation of protopine is qualitatively different in female and male mice after a single dose (29).

## Conclusion

In conclusion, the findings from this study demonstrated that protopine is the primary toxic component in *M. cordata*, with an i.g. LD_50_ of 313.10 mg/kg in mice. Protopine exposure induced toxicological effects on various organs in mice. Based on the mouse behavioral changes, organ weight (absolute and relative) changes, autopsy, and H&E experiments, the liver and brain were the seriously damaged organs. The results suggest that medicinal plants containing protopine may be harmful to humans and animals. This information will be valuable for further assessment and evaluation of the toxic characteristics of protopine.

## Data Availability Statement

The original contributions presented in the study are included in the article/supplementary material, further inquiries can be directed to the corresponding author/s.

## Ethics Statement

The animal study was reviewed and approved by the Northeast Agricultural University Animal Ethics Committee and the approved protocol number: SRM-06.

## Author Contributions

WH, FY, and WL carried out extraction and bioactivity-guided fractionation of *M. cordata* experiments. WH, LG, and LA did the acute toxicity animal experiment. WH and ZS participated in the design and wrote the manuscript. ZS and CG revised the manuscript. All authors contributed to the article and approved the submitted version.

## Funding

This research was supported by National Natural Science Foundation of China (Grant No. 31572559) and Academic Backbone Project of Northeast Agricultural University (Grant No. 16XG16). The open access publication fees, from National Natural Science Foundation of China (Grant No. 31572559).

## Conflict of Interest

The authors declare that the research was conducted in the absence of any commercial or financial relationships that could be construed as a potential conflict of interest.

## Publisher's Note

All claims expressed in this article are solely those of the authors and do not necessarily represent those of their affiliated organizations, or those of the publisher, the editors and the reviewers. Any product that may be evaluated in this article, or claim that may be made by its manufacturer, is not guaranteed or endorsed by the publisher.
